# Existential Well-Being in Nature: A Cross-Cultural and Descriptive Phenomenological Approach

**DOI:** 10.1007/s10912-024-09846-0

**Published:** 2024-04-13

**Authors:** Børge Baklien, Marthoenis Marthoenis, Miranda Thurston

**Affiliations:** 1 Faculty of Social and Health Sciences, Inland Norway University of Applied Sciences, Hamarveien 26, 2418 Elverum, Norway; 2 Department of Psychiatry and Mental Health Nursing, Universitas Syiah Kuala, Banda Aceh, Indonesia

**Keywords:** Nature, Applied phenomenology, Ecological public health, Well-being

## Abstract

Exploring the putative role of nature in human well-being has typically been operationalized and measured within a quantitative paradigm of research. However, such approaches are limited in the extent to which they can capture the full range of how natural experiences support well-being. The aim of the study was to explore personal experiences in nature and consider how they might be important to human health and well-being. Based on a descriptive phenomenological analysis of fifty descriptions of memorable moments in nature from England, Indonesia, and Norway, our findings illustrate a common structure presented under three themes: 1. serenity that gives rise to a growing awareness of how the body is stimulated by the senses; 2. admiration and appreciation for the sensation of beauty; 3. an emerging sense of togetherness and deep emotional bonding. The findings are discussed using the concepts of ecological time and the ecological body, which foreground being in nature as constituted as an interdependent and dynamic human process. We conclude by understanding well-being in terms of human responsiveness to their surroundings and thus as rooted in the human condition.

## Introduction

Societal shifts over several decades continue to reconfigure people’s relationship with the natural environment, shifts which have been amplified in recent decades. The migration of people to cities, alongside the attendant urbanization, has distanced many people from nature, a trend that is set to continue (Soga and Gaston 2023). In the same vein, the expansion of technology relating to the consumption of media has contributed to the rise in sedentary lifestyles that are increasingly home-based (Woessner et al. 2021). A consequence of these trends is not just that many people have been physically repositioned away from the natural environment (Pritchard et al. 2020; Nisbet, Shaw, and Lachance 2020; Rosa, Profice, and Collado 2018) but that our collective imagination and cultural conversations about nature have also declined since the 1950s (Kesebir and Kesebir 2017). At the same time, however, there has been a resurgence of interest in human-nature relations from both the public health and the environment fields. For example, there is now a substantial body of evidence that supports the long-standing view that being in the natural environment promotes mental health (in terms of psychological struggles and challenges) and well-being (in the sense of thriving and/or flourishing as conceptualized by Westerhof and Keyes [2010]) (Zylstra et al. 2014; Ives et al. 2017; Soga and Gaston 2016; Richardson, Passmore, et al. 2021; Baxter and Pelletier 2019; Hurly and Walker 2019). There is also an emerging body of evidence that indicates that enhancing people’s connection with nature can increase the likelihood that they support biodiversity and pro-nature policies and actions (Soga and Gaston 2023). While many researchers have tended to rely on biomedical, reductionist approaches to the study of human connection with nature, we view this emerging body of work as raising some important ecological questions about purpose and meaning in humans’ relations with nature. Thus, we position our study within the health humanities field, contextualizing wellbeing as rooted in the human condition as circumscribed by modernity (George et al. 2023), of which relations with nature are a part. Our specific interest and departure point is to understand how people perceive and experience being in nature. We use a qualitative phenomenological approach to generate rich and detailed accounts that can give insight into the existential dimensions of human-nature relations. We start by reviewing the extant research in the field to provide a rationale for our approach.

## Conceptualizing Human-Nature Relations Phenomenologically

Much of the empirical research to date—for example, in the field of environmental psychology—has tended to rely on a quantitative paradigm to describe and explain human-nature relationships. This has involved measuring contact with nature—conceptualized as an “exposure” to, for example, the sea, forest, or countryside—and using various psychometric scales to measure various psychological and health outcomes (Muhr 2020; Ives et al. 2017; Zylstra et al. 2014; Barragan-Jason et al. 2023). This type of research has given rise to questions of how much contact with nature is needed if benefits are to be realized, in keeping with a quantifiable “dose-response” framing of “exposure” to nature. For example, visiting nature more than once a week or watching nature documentaries has been linked to health and eudaimonic well-being (relating to a meaningful life in terms of fulfillment and purpose, again as conceptualized by Westerhof and Keyes [2010; Martin et al. 2020]), while spending two hours a week in nature has been related to better health and well-being (White et al. 2019). The theoretical underpinning of many of these studies has largely been in terms of cognition (for example, cognitive restoration in Attention Restoration Theory [Kaplan 1995] and affective, cognitive, and psychological restorative processes in Stress Recovery Theory [Ulrich 1984]). Likewise, being attuned to natural beauty has been related to affect regulation and well-being outcomes such as happiness through cognitive mechanisms (Richardson and McEwan 2018). This type of empirical and theoretically driven research has given rise to a broad consensus across the natural, social, and health sciences that being connected to nature can have an impact on various dimensions of mental health, including cognitive function, emotional and subjective wellbeing, psychological connectedness, and, physical health (Bratman, Daily, et al. 2015; Martin et al. 2020; Bratman, Anderson, et al. 2019; Olivos and Clayton 2017). Consequently, nature has been increasingly viewed as having the potential to be used in the treatment of depression, stress, negative emotions, cognitive challenges, and anxiety (Ulrich 1984; Martyn and Brymer 2016; Marselle, Irvine, and Warber 2014; Mayer et al. 2009). Thus, Barton and Pretty (2010) conclude that the question is no longer whether connectedness to nature improves health but rather how nature is beneficial and how it might be utilized in health promotion.

Framing human-nature relations in this way crowds out broader understandings of human experience that are better viewed as holistic, dynamic, and contextualized. From an empirical point of view, quantitative studies based on a cross-sectional study design can only capture brief and localized episodes and cannot explore how human-nature relations might be understood as cumulative (Krekel and MacKerron 2020). Moreover, they tend to fragment and reduce human experiences to specific variables, captured at one point in time and linked statistically to specific impacts. In recent years, there has been an emphasis in the field on the development of measurement tools (Restall and Conrad 2015) relating to three interacting dimensions: cognitive, affective, and experiential connections (Muhr 2020). This implies that the concept of “well-being” is a construct that can be measured rather than as an experience that can be expressed in more complex and multilayered ways. Similarly, theoretical departure points also contribute to narrowing conceptualizations of human-nature relations. For example, some studies have framed nature as a psychological human need, the satisfaction of which is viewed as a pre-requisite for experiencing well-being (Baxter and Pelletier 2019; Hurly and Walker 2019). In the same vein, the biophilia hypothesis is based on the premise that humans have an innate bond with nature rooted in our evolutionary history, and thus, humans, by instinct, are attracted to and seek out nature (Wilson 1984; Selinske, Harrison, and Simmons 2023). While this body of research is extensive and informative, its tendency to fragment and narrow human experience and subjectivity leaves unanswered how we can better understand human experience in terms of the purposes and meaning of being in nature (van Spijk 2015). Thus, as far as we can see, it is still rather unclear what constitutes “experience in nature” (Hartig et al. 2014, 209; Trigwell, Francis, and Bagot 2014) and how this might be related to well-being.

Philosophical approaches such as eco-psychology and eco-phenomenology may offer a helpful entry point for understanding connectedness with nature in experiential terms (Abram 1997; Toadvine 2005; Fisher 2013; Vakoch and Castrillón 2014). This work reveals that nature can be experienced as a sense of peacefulness and spiritual well-being (Heintzman 2009), as human well-being (Schweitzer 2021), and as a peak transformative experience (Naor and Mayseless 2020). At a more descriptive level, nature is appreciated for its scenic values (Dorwart, Moore, and Leung 2009) and as a setting for recreation (Haaland and Tønnessen 2022), such as when families hike together in nature (Baklien, Ytterhus, and Bongaardt 2016), while children express their love for nature (Kalvaitis and Monhardt 2015). This body of research suggests that phenomenology offers a potentially fruitful way of generating insights that can help us capture a wider breadth of meaningful experiences in nature as well as illuminate how such experiences might support well-being (Schweitzer 2021). In other words, phenomenological approaches ask *how* we experience moments in the natural environment as health and well-being (Grant and Pollard 2022). Because phenomenological research explicitly attempts to understand the world of another by framing experiences as they relate to the whole person situated within the context of, in this case, nature, it potentially addresses the limitations of the extant research outlined above. In this regard, the bodily aspects of experiencing nature and how these relate to meanings and purposes—and, thus, well-being—have been little explored in previous research, which has tended to separate the senses and feelings from the body. Phenomenology unites the corporeal body with the lived body (Orphanidou, Kadianaki, and O’Connor 2023), avoiding objectification and focusing on understanding embodied experience as one’s experience: while the “corporeal body senses … the lived body understands” (Rentmeester, Bake, and Riemer 2022, 445). Informed by the health humanities field (see, for example, Coope 2021; George et al. 2023; Valtonen and Lewis 2023), we conceptualize human connection with nature and well-being phenomenologically and, thus, in existential terms—that is to say, in terms of human everyday lived existence. Thus, we explore, describe, and clarify how well-being is lived within an ecological and cross-cultural context. This offers the prospect of gaining a better understanding of how nature might be beneficial and might be used within an ecological public health perspective (Lang and Rayner 2012).

## Methods

In choosing phenomenology as an approach, we give emphasis to people’s lived experience and subjectivity. To date, research on connectedness to nature has largely been undertaken in post-industrial Anglo-Saxon countries (Ives et al. 2017; Restall and Conrad 2015). Our research takes a cross-cultural perspective and explores participants’ experiences of being in nature in three diverse countries, namely, Indonesia, Norway, and England.

### Approach

Students from England, Indonesia, and Norway were asked to write down descriptions of a memorable moment they had experienced in nature. The aim was to generate descriptions of their lived experience rather than analytical accounts of their experience. Thus, the purpose of using this method was to generate descriptions of subjective experiences of nature, not opinions, beliefs, or accounts about experiences (van Manen 2016, 299). Due to our experiential approach, a descriptive phenomenological method was chosen to allow a deeper insight into memorable moments in nature by staying close to the experience.

The phenomenological method involves adopting an “attentiveness and wonder” towards the world and making an effort to relearn how to look at the world. As such, our attention was on how the world was present to us (Merleau-Ponty 2012). In the phenomenological literature, this is called bracketing and may be understood as an act of suspending the tendency to objectify the world (van Manen and van Manen 2021, 13). In other words, the researcher tries to put aside theoretical or ideological assumptions during data collection and analysis by withholding any existential positing of the truth and how it is classified in the objective world. Rather, the focus becomes the phenomenon and meaning and how they appear to the research participants.

However, as qualitative researchers, we recognize that we are not neutral observers; we are narrators and meaning-makers inspired by and involved in the literature and the relation between phenomenology and nature. Through our data analysis and presentation of findings, we engage in storytelling by weaving our students’ descriptions into a narrative about meaningful moments in nature. Thus, at some level, we are constituting the meaning we find in our data (Churchill 2022, 48,73) in order to provide an interpretivist account. Bracketing out was an ongoing process of seeking to challenge taken-for-granted assumptions and ways of viewing the world by questioning how we understood the descriptions from our students.

### Participants and Data Collection

In common with other qualitative methods, phenomenological research works with small samples rather than representative samples as it is primarily concerned with a rich, detailed account of individual experience. The backdrop to this study was an established research collaboration between Norway, England, and Indonesia and our shared interest in nature-based services in mental health, especially with regard to how being in nature may be experienced as well-being. Given the differences in landscape between the countries, experiences were expressed in relation to the sand and sea, the mountains and fjords, as well as the rolling hills of more gentle countryside. We invited university students in diverse master’s programs from England, Norway, and Indonesia during normal class time to participate in the study. All students who were present on the day the research took place were invited to participate, regardless of age, gender, or socioeconomic background. Overall, eight students from England, 20 from Norway, and 22 from Indonesia participated. Given our descriptive approach, we started by asking research participants to write experiential descriptions of memorable moments in nature. We gave the participants a simple instruction: “Please describe a moment in nature that has meant something special to you or as you remember it.” Each student wrote their experiential description on their computer and sent it to the researchers. Descriptions varied in length from half a page to two pages. All descriptions written in Indonesian and Norwegian were translated into English by professional translators before the data analysis. In each of the three countries, the appropriate ethical review processes were followed. Participation was by written informed consent.

### Data Analysis

Fifty descriptions from half a page to two pages were analyzed using Giorgi’s (2009) five-step descriptive method. The findings in the descriptive phenomenological method are the meaning structure of the phenomenon, which is expressed as a consistent statement. In the analysis, we initially differentiated between the three countries and thus went through all five steps three times. However, comparing the meaning structure of these three countries, they were similar in how the sensual body experiences being in nature. Thus, we integrated the three meaning structures into one, which we present in the findings.

The first step in the analysis was an initial reading of all the transcribed descriptions, which gave a sense of the whole experience.

In step two, we tried to “bracket” or “take out of play” our preconceptions about the phenomenon to lessen the influence of our knowledge, beliefs, and theoretical assumptions to prevent us as researchers from mistaking our beliefs as scientific findings (Churchill 2022, 83). Using this kind of thinking, all transcriptions were read with an effort to apply a phenomenological attitude to develop a preliminary sense of the whole and some idea of how the description proceeds and ends (Giorgi 2018, 98).

In step three, we re-read the descriptions, dividing them into smaller manageable parts or meaning units by setting a slash when we experienced a transition in the meaning. Such meaning units can be a sentence or a paragraph and allow for a more detailed focus later in step four. We modified the descriptions into third-person expressions by replacing* I* with *P* (participant) and with she or him to be better able to see the differences between the individual and the phenomenon (Giorgi 2009).

The fourth step was a process called imaginative variation, in which the researcher tried out different formulations of each meaning unit at various levels of generalization into a transformed meaning unit. This involved finding ways of expressing the implicit meaning in a more direct and explicit form by extracting and reflecting upon each of the delineated meaning units without using theoretical terminology. All the meaning units were re-read to formulate what they reveal about the participants’ experience in meaningful moments. No coding was undertaken; rather, all the meaning units were interrogated for their relevance and transformed to highlight the psychological meaning attached to them. This transformation process step lies at the heart of the method of phenomenological descriptive analysis, where the psychological meaning must be “detected, drawn out and elaborated” (Giorgi 2009, 131). Table 1 gives an example of this transformation process.

**Table 1:** Example of the process from transcript to transformed meaning unit

*Transcript*	*Third person meaning unit*	*Transformed meaning unit*
When I look at the beach, I feel calm and cool, as if all the burdens of my mind have temporarily disappeared. The blue color of the sea is very beautiful and soothing to the eyes, the sound of the waves and wind can distract my mind.	When P looks at the beach, P feels calm and cool, as if all the burdens of her mind have temporarily disappeared. The blue color of the sea is very beautiful and soothing to the eyes, the sound of the waves and wind can distract her mind.	The sensation of the beach makes P feel at ease as if all her worries and stressful future assignments temporally disappear from her mind. She became aware of what was going on in the moment instead of burdens. She notices her senses are immersed in the present and how the beautiful blue color of the sea, the sound of the waves, and the wind direct her mind to the present.

In step five, and based on the transformed meaning units, we synthesized all the descriptions into the general meaning structure. We did this by synthesizing the transformed meaning units into a consistent statement to give the general meaning structure of the experience of memorable moments in nature, thus capturing the experience across participants. The structure must be found in all the descriptions to be part of the essence of the experience of the phenomenon. According to Giorgi (2009, 200), it is not “a matter of simply listing the meaning units together but to bring a holistic perspective on them.” In this way, all the participants’ experiences of the phenomenon were included by reviewing all the transformed meaning units and comparing and contrasting them to decide which ones were necessary for describing the general meaning structure. In essence, in this step, we tried to determine what was essential to the description by moving from the individual to the general level.

## Findings

Through descriptive phenomenological analysis, we revealed a general meaning structure of the experience of meaningful moments in nature for each country. By comparing the three meaning structures, we concluded that, despite some cultural differences in how to be out in the natural environment between the countries, the meaning structures were similar. The one noticeable point of divergence was that the Norwegians tended to enjoy such moments with others but also alone. In the following, we first present the common essential meaning structure and then clarify it.

Meaning structure for the phenomenon of memorable moments in nature.

In situations where memorable moments are experienced, P feels present where she is and does not think about the rest of the world, her worries, or time. Instead, she feels how she becomes drawn into the view, the smells, the sounds, and how the air, water, and ground touch her skin. P’s body feels peaceful, and she lets go of her concerns which brings a sense of relief. P experiences the moment as if a veil of thoughts and tasks that occupy her in everyday life is lifted and she feels how her senses become aroused by the surroundings here and now. In such a moment, P becomes aware of how the beauty in nature touches her and how she feels some deep emotional connection and togetherness with those who are with her and with the more-than-human world. It is as if the arousal of her senses is related to “letting go” of thoughts and worries from everyday life, which gives the body and mind space to open up further and deepen the sensual and emotional experience of being in nature with no distraction.

### The Serenity that Gives Rise to a Growing Awareness of How the Body is Stimulated by the Senses

When memorable moments emerge in an aesthetic landscape of serenity, it is felt and experienced as letting go of the stresses and strains in everyday life, which brings a sense of relief. This peacefulness is transmitted to the body, and it feels like “letting go” in such a way that thoughts and worries from everyday life are released from the mind. Without these distractions, outer peacefulness becomes or gives rise to an inner peacefulness that enables the senses to be aroused. As such, the moment evokes a sensual embodied awareness where the present becomes primary while the rest of the world—worries, school, work, or time—recedes into the background. When compared with their everyday life, they become aware of how they are “released from all everyday worries” and more open to seeing the details and richness in their surroundings. A student from England describes how she became aware:

The path we took was well-trodden, compiled of small pebbles, and made the ground slip and compress as we walked making soft crackling sounds combined with the sounds of waves which I found soothing. I had just finished a long and stressful final year at university, having only completed my undergraduate a week or two before. It was nice to be active and free after being constrained to a library for months.

Without all the distractions and constraints of everyday life, they experience a sense of presence that is mentally refreshing. The serenity allows them to become more aware of how their bodily senses are stimulated. The temperature, the sight of the water, the feel of the ground, the various sounds and smells envelope them in such a moment, and a deeply emotional sense of serenity appears. As a student from Indonesia describes a moment on a hiking trip:

Along the way [to the top of a mountain] I paid attention to whatever was coming. I felt peaceful when I saw a sight that rewards my eyes, and comfortable with the cool air that I feel, the breeze that always makes me not want to go home. Supported by a whispered voice of leaves that did not allow me to leave. All of that makes my body very relaxed. This feeling of peace and comfort is like nothing can disturb it.

In such a moment, nature is in various ways beautiful; however, what they specifically describe as beautiful varies between the countries—from Indonesia with the beaches, the rice fields, green mountains, and the cool breeze, in England with the countryside, national parks, cliffs, and the beaches; and, in Norway with the mountains, forest, sea, and lakes. Nevertheless, they all bring forth their bodily senses, which they draw on to describe the setting and the evocation of serenity and delight of being present in the moment. More specifically, they feel relaxed and attentive at the same time and, as such, feel a bodily refreshment. By feeling relaxed, they become alert and open to perceiving their surroundings, and by being attentive, they notice such details as the color of the leaves or the silence. They are attuned to themselves and their surroundings and open their senses, letting the landscape “enter in.” It is an immediately embodied response to the shapes and natural forces around them. This student from Norway describes a moment in the forest in poetic language:

Then a light drizzle began to whisper through the wood. The leaves on the birch trees were still almost completely green, even though they had noticeably faded. Only here and there were some red and yellow leaves twinkling in the sun when the rays suddenly slid down through the dense network of thin branches just washed by the falling rain. Not a single bird could be heard; they had all sought shelter and calm. Just the occasional cheerful sound of a titmouse could be heard like a tinkling bell through the birch grove.

### Admiration and Appreciation of the Sensation of Beauty

Many of the descriptions are similarly poetic rather than explanatory. The experience of the sunset, the ocean, the rice field, the mountains, the chilly air, and the green forest is aesthetic and evokes emotions. For example, the rice field is not just food or work; it is something more; it is experienced in a deeply moving way where the whole body is active and absorbed in the beauty, as this Indonesian student describes.

I live at home in the middle of a wide expanse of rice fields. The rice fields are green and soothing. Every morning, the rice fields are foggy. During the day, there are a lot of breezes. And in the afternoon the rice fields look very beautiful because of the added reflection of the sunset. Especially during the harvest season, the rice began to turn yellow, and the rice fields were full. This is a beautiful sight to the eye. Every morning I like to go out to see the fog covering the rice fields. It feels cool and serene, except for the sound of birds singing. Every morning, farmers started arriving and descending into the fields. There you can often hear the sound of many iron cans tied to the rope and then shaken to keep the birds from eating the rice.

Such meaningful moments happen when the body feels undistracted by the routine of everyday life and especially attentive to the surroundings, which they thus perceive as particularly beautiful. In such a moment, which can last for seconds or persist for some hours, they feel a sense of warmth, admiration, joy, and appreciation for what they are experiencing. They highlight the sensuousness in their descriptions, such as the sound of the leaves, the smell of the ocean, the feel of the cooling wind, and the view from a mountain. Becoming attentive brings forth an appreciation of something beautiful, which rests on an emotional and bodily sense of being touched by it.

Although what is perceived as beautiful may vary, the experience is described in a similar vein. In Norway, beauty is described as when the sunshine colors the natural environment, the firmament on a clear winter night, embraced by the forest and the stunning view from the mountains. This student captures how such moments give them a warm, deeply moving feeling.

As we move on up, our view changes to lush hills with autumnal trees, while lakes reflect nature in all its glory and look like paintings as they shimmer in the calm landscape with the sun shining between the clouds, creating a magical image of our country. I feel touched by how beautiful Norway is in its autumn colors. I get a nice warm sentimental feeling in my chest and feel very lucky and privileged to be able to have such an experience.

In England, the beauty is described in similar ways with the stunning view of the countryside, the cliffs, the beaches, the ocean, the waterfalls in Wales, or the spectacular view when hiking around in the world that brings forth a deep sense of awe imprinting this as a long-lasting memorable moment. It is a sense of gratefulness, as this student describes:

Throughout the pleasure ride there were moments when I was left by myself as my partner and horse galloped off into fields. During this time, it was nice to reflect on how lucky I feel to have access to such beautiful scenery, environments, and a horse. Something that I have always wanted in life was happening at that very moment, and as cliché, as it sounds, I think it will always be unforgettable. It was peaceful to just stand there and take in the scenery and simply be present, happy, and grateful, even though I was giving appreciation to one particular person.

In Indonesia, the aesthetic is described similarly with the beaches that offer extraordinary beauty, the rice fields, and the mountains wrapped in a green carpet of plants and trees, which brings forth a sense of gratefulness. As this student from Indonesia describes, it is as if the peaceful movement of the sea, the chilling breeze, and the salty smell all together capture the mind and body.

When looking at the beach I feel calm and cool, as if all the burdens of my mind have temporarily disappeared. The blue color of the sea is very beautiful and soothing to the eyes, and the sound of the waves and the wind fills my mind. The beauty, smelling the salty aroma of the sea, and hearing the waves breaking on the shore, made my heart and mind calm again. It evokes gratefulness to Allah the creator for the beauty of nature.

### An Emerging Sense of Togetherness and Deep Emotional Bonding

In memorable moments, they are seldom alone. However, the descriptions refer to their emotional attachment to being in nature. Often, they are with family, friends, or their lover, and in such moments, they appreciate their feelings towards them. They start to see the people around them more clearly and with affection. Feeling serene and letting go of tensions brings greater visibility to other people while, at the same time, sharing the experience with others creates an emotional sense of togetherness. As this student from England describes, during such undisturbed moments in nature, her relation to her partner is intimate and experienced as closeness.

I was able to see my partner properly without work or stress crossing my mind and I was fully immersed in the “moment.” We went “shell collecting,” investigated the rock pools for creatures along the shore, and childishly pushed each other in the water and quicksand—it was a freeing experience to just be silly for a while. We laughed and joked as well as had more serious conversations reaffirming our relationship and how we saw our future together.

Such a moment in nature gave rise to a deepening of feelings towards others they were with and a strengthening of emotional bonds that could last for a lifetime. In such a moment, they were touched by the beauty of something bigger than themselves, which they could share with somebody close. Sharing these kinds of meaningful experiences amplified the enjoyment of the situation, and a deep feeling of gratitude for the experience and a sense of bonding emerged. Sharing such moments with persons they were attached to, as this mother from Norway describes, contributed to a sense of deep emotional bonding.

We came to a small pond and the sight almost took my breath away. The sky was reflected in the water with its pink clouds and the bluish twilight. It was just like a painting. I felt a shiver of happiness run through my body. Partly because of the beautiful view, but also because I could share that moment with my family.

It is not only families or lovers that feel such emotional bonds. Sharing such moments of awareness and serenity with friends, as this description from Indonesia illustrates, a sense of ease and togetherness emerge.

During the climbing trip from the foot of the mountain, it was very cool with the sky that was getting dark and a little drizzle. We reached the top at night, then my friends and I built a tent together and cooked around the tent. When you are at the top with friends is the most exciting time.

It is striking how such moments created memories that linked pleasant feelings of the environment with sound, smell, and temperature, together with pleasant feelings of just being with others. For example, this description from England illuminates the feeling of sitting under a blanket with someone on the beach on a windy day.

Reasonably strong wind, I could see the sand moving across the surface of the beach. Seeing the sand blow onto our shoes and seep into our socks. Taste the sea salt in your mouth, and feel the sand hitting your face. See the beach grass bending over sideways due to the force of the wind. Seaweed rolled across the sand gathering debris as it went. Pools of seawater with surface rippling as buffeted by the wind. Laughing, giggling, huddled together very close to keeping warm. Sitting on one blanket and wrapped up in another tartan, a woolen blanket that smells slightly of an old damp Welsh cottage. Hair flying about, whipping you in the face. Holding on tight to the blanket so it didn’t get pulled from us. Occasionally burying our heads inside the blanket to get a break from the salty wind.

The description conveys a closeness that requires few words to be spoken. Together, they have a shared world, experiencing the same things, sometimes teasing each other, and, at other times, they become occupied by focusing deeply on the things around them. The value is just to be together and feel close to those they are with. In the same manner, this student from Norway describes how such moments are about a sense of togetherness:

These were magical experiences for us and the children. We made a bonfire by the river, as it was often a bit dark and chilly outdoors. The bonfire gave us warmth and light, we could feel its heat and we grilled sausages to fill our stomachs. The children shifted between fishing and sitting by the bonfire, and sometimes they played a bit in the water as well. If they caught a fish, their happiness was indescribable. They almost jumped for joy, cheered with their whole bodies, smiled from ear to ear, and were just so happy and proud.

The moment could also involve contemplation and wonder about life because being together in nature created space for these deeper kinds of thoughts. They involved each other in their reflections and thoughts about life. Such moments were felt to be deeply meaningful, and they connected at an emotional level to others and themselves. Sharing personal thoughts about life gave rise to feelings of bonding, which contributed to emotionally forging their relationship. This description from England describes how nature almost brings out situations that bring forth closeness and a sense of emotional bonding.

The sun was still awake, but it was getting to the point where it was almost time for it to start descending. We got to the top and we all decided to take a break, eat, and chat. I will always remember this day because as we were watching the forest and sun beyond us, we started talking about our future goals and aspirations. It was almost like nature itself was bringing it out of us. Personally, I felt at inner peace and had forgotten all that was wrong with the world. Hear the wind whistling, seagulls shrieking, trying to fly to safety.

## Discussion

By exploring memorable moments in nature, our findings support the overall conclusion of previous research that “contact” with nature can contribute to well-being. However, using a qualitative phenomenological approach to conceptualize human beings holistically and contextually has allowed us to present new ways of understanding such a relationship. Based on our empirical findings, here we discuss our preliminary theoretical ideas to account for how nature supports a sense of well-being, at least in the here and now. We use the term *ecological* to foreground the notion that being in nature is constituted as an interdependent and dynamic human process. We thus develop our theoretical account of how being in nature contributes to well-being using two interrelated ideas: ecological time and the ecological body, both of which we explain further below. Importantly, this theoretical account is relational in the sense that being in nature is juxtaposed with being in everyday life, which is likely to be quite different, particularly in terms of being disconnected from nature, especially immersive experiences in nature.

### Nature and Well-Being: Ecological Time

As we outlined earlier, societal shifts over several decades have contributed to a structuring of people’s daily lives that are harried and in which their rational selves are emphasized at work and home. Life thus becomes routinized through obligations and other distractions, a consequence of which is that senses and emotions are often constrained (Elias and Dunning 1986). Our opportunities for mental refreshment that are intensely meaningful and give us purpose may be few (Jackson 2011). Our findings suggest that being in nature stands out as being different in this regard because being in nature can lead to purpose and meaning on which our well-being pivots.

However, our findings suggest that being in nature is experienced subjectively rather than objectively as if it exists independently of human experience. The English author and poet Thomas Hardy, writing in the nineteenth century about his home county of Dorset in the south of England, said that “experience is as to intensity and not to duration” (Hardy 2008). Similarly, Merleau-Ponty (2002) compares time to the perception of the moving landscape:

If the observer sits in a boat and is carried by the current, we may say that he is moving downstream toward his future, but the future lies in the new landscapes which await him at the estuary, and the course of time is no longer the stream itself: it is the landscape as it rolls by for the moving observer. Time is, therefore, not a real process, not an actual succession that I am content to record. It arises from *my* relation to things. (Merleau-Ponty 2002, 478; original italics)

We interpret our findings as revealing that people experience time somewhat differently when in nature because of the intensity of their experiences, as Hardy reminds us. This gave rise to a feeling of being in the “present” such that time seemed to stand still. We might view this as being in nature’s unfolding rhythms in what is called “soft fascination” (Kaplan 1995). Put another way, the complexity of everyday life is replaced by simplicity in nature (Baklien, Bongaardt, and Ytterhus 2016). Intense experiences that are suspended in time displace the stresses and strains experienced in everyday life, which thus recede to the background and become merely a “storm on the horizon.” Experiencing time in this way gives space to feel alive and refreshed. In other words, being in nature is a way of circumventing what has been called the “social malaise associated with modernity” (George et al. 2023), and it is this that gives rise to expressions of well-being.

### Nature and Well-Being: The Ecological Body

The attraction to nature’s beauty and aesthetics is well-documented and a central dimension to understanding well-being (Kaplan 1987; Ulrich 1984; Richardson and McEwan 2018). However, what a health humanities perspective adds is the way it positions well-being in terms of human *responsiveness* to surroundings and, therefore, as rooted in the human condition (George et al. 2023). This means that understanding how people interpret and create meaning in their experiences is central to understanding well-being (Valtonen and Lewis 2023). Such a perspective opens up possibilities for providing a more multifaceted understanding of well-being than has hitherto been the case. Our findings reveal that human responsiveness to being in nature primarily involves the evocation of the senses—not just sight, but sound and touch, too—alongside an enhancement of the awareness of those with whom they are. Responsiveness was also related to surges of a range of emotions. Whilst being in nature is often viewed as leading to calmness, we view these responses as giving rise to feelings of mental refreshment and heightened awareness; not only have they “let go” of thoughts and worries from everyday life that typically distract their attention, in doing so they have space to be open to nature and what it offers. When human beings allow their senses to be captured by the surrounding nature and its beauty, it enriches the present. Critically, we also interpret responsiveness as having a bodily dimension—such that being in nature is an embodied experience. Thus, in common with others in the mental health field (see, for example, Orphanidou, Kadianaki, and O’Connor 2023, 516), we view the body as an active agent in shaping experiences and as “critical in ascribing meaning to … experiences.”

The concept of the ecological body supports this form of theorization in the sense of an “immanent, co-creating, moving body: a body constantly becoming within a changing environment, where the body and the spaces in between and around bodies are considered as equally dynamic” (Reeve 2008). Thus, the ecological body moves and sensually experiences being close to nature rather than as an objectified unit acting on nature as a static entity. Perception of nature is thus viewed as a lived dynamic between the body and the world mediated through the senses: we immediately hear the soft wetness of the ocean, see the hardness of a stone, and smell the dryness in a dessert as nature speaks to all our senses at the same time (Merleau-Ponty 2012, 238). In this interpretation, the ecological body responds to the perception of communal sharing with nature through emotions. Sensing nature alone or with friends, families, or loved ones gives rise to pleasant sensations of warmth from the inside. Such emotion can be described as “*moved, touched, stirred, heart-warmed*, experiences *rapture*, gets *the feels*, or has *tender* feelings,” which are captured in the concept of “kama muta,” which conveys how moments in nature are intensified and when the sense of being moved is strong, it becomes memorable (Fiske 2019, xviii; original italics). In our findings, while it varied how strongly emotions were experienced, they were an essential part of how memorable moments were experienced. When the richness of the natural surroundings “assaults” the senses and gives rise to an embodied sense of transformation, then people are drawn out of their usual way of life into a sense of mental uplifting and refreshment.

In everyday life, experience is often characterized by an absence of bodily awareness (Csordas and Harwood 1994). Our analysis illustrates that bodily awareness emerges when human beings practically engage with the world, and the strains and stresses of everyday life recede into the background. Thus, when in nature, our students described the sensuous lived body “which we are” (Van den Berg 1972, 54). Furthermore, we interpret experiences in nature as relating to the whole social situation wherein we find ourselves without everyday distractions. The embodied response to nature creates an existential sense of well-being, that is to say, of “letting be.”

Ostensibly, mindfulness may seem to capture the experience of connectedness to nature as “being attentively aware of what is taking place in the present” (Brown and Ryan, quoted in Howell et al. 2011, 167; Schutte and Malouff 2018). However, mindfulness as a concept has limited explanatory power in terms of our findings, given that it individualizes the capacity to sense, feel, and notice what is going on within oneself. We interpret our findings as pivoting on the concept of responsiveness and a more expansive view of the mind, the “mind as released from the confines of the body into our relational patterns of engagement” (McNamee 2021). Thus, sensory pathways are involved in the surroundings (Bateson 2000). The beach, the countryside, and the mountain give rise to different sensory pathways and engagement with the environment. It is a movement into serenity with an awareness of the possibilities of engaging with others and nature with a sense of vitality or existential well-being (e.g., Dahlberg, Todres, and Galvin 2009). It supports the argument that to become sensitive to the beauty in nature it is not just about visiting or exposure to nature, but rather, one needs to engage in an affective relationship if well-being is to emerge (Richardson and McEwan 2018; Richardson, Hamlin, et al. 2022). Moreover, we conclude that well-being is experienced within this process when the body, the mind, and the world become aligned as though they were three characters participating in conversation together—“three notes suddenly making a chord” (Solnit 2001, 5).

## Conclusion

Although well-being is a contested concept, there is some consensus that it is best viewed as an existential construct related to the purpose and meaning in life (Ryff 2014). We conclude that being in nature can give rise to existential experiences that contribute to a sense of well-being. Furthermore, our paper contributes to developing a better understanding of the ecological dimensions of well-being (Coope 2021). Notably, qualitative research underpinned by phenomenology can inductively inform how we understand well-being. It highlights how studying people’s experiences can contribute to developing a more complex, multifaceted understanding of well-being, which unfolds in everyday life. Quantitative research, on the other hand, conceptualizes contact with nature in terms of exposure, dose, and effects, overlooking the ecological—that is to say, interactive character—of being in nature, during which all the senses (not just the visual) play a part in generating an overall experience (Conniff and Craig 2016). At the same time, quantitative research tends to fragment these sensory experiences into measurable variables that objectify phenomena as outside of human meaning and, in so doing, obscure the multiple pathways and mechanisms that might occur in the same natural environment (Bratman, Anderson, et al. 2019). However, our conclusions need to be interpreted with caution, given the limitations of the qualitative phenomenological approach used in this study. While we offer an interpretation of our participants’ perspectives that we believe to be trustworthy (Lincoln and Guba 1985), the methodology has the potential for various forms of bias that include our own preferences and expectations regarding the phenomenon, which may not have been fully bracketed out. There are also limitations relating to the composition of the sample and the countries in which participants resided. Future research can test the extent to which the findings and interpretations might be valid in other contexts. Further research might also combine and integrate quantitative and qualitative methods through a process of triangulation, which might clarify which dimensions of existential experience are closely related to experiences in nature. In addition, future studies could more fully explore how sharing experiences in nature with family and friends might contribute to individual and group existential experiences, something that was apparent in our data but not fully explored. Walking and talking methodologies would be a useful way of generating communal stories, while quantitative approaches could clarify which social and interpersonal variables are associated with well-being. Thus, the findings from the current study can be used to inform better ways of studying human contact with nature. In the contemporary public health landscape, this seems important given that experiences of nature may well be restorative for mental health and well-being.
